# Limited and Mixed Evidence for System-Sanctioned Change to Protect the Environment: A Replication Study

**DOI:** 10.5334/irsp.871

**Published:** 2024-08-30

**Authors:** Inkuk Kim, Samantha K. Stanley, Kirsti M. Jylhä, Nic Badullovich

**Affiliations:** 1 School of Psychology, Victoria University of Wellington, New Zealand; 2 UNSW Institute for Climate Risk & Response, University of New South Wales, Australia; 3 School of Psychology, University of New South Wales, Australia; 4 School of Medicine and Psychology, Australian National University, Australia; 5 Institute for Futures Studies, Sweden; 6 George Mason University, Center for Climate Change Communication, Fairfax, Virginia, United States; 7 Crawford School of Public Policy, Australian National University, Australia

**Keywords:** system justification, system dependence, system preservation, framing study, pro-environmental behaviour

## Abstract

Feygina and colleagues (2010, Study 3) reported that people who prefer the status quo can be encouraged towards pro-environmental responses when environmental protection is framed as protecting the current way of life. We report a preregistered close replication and extension of this work (*N* = 567). When all participants are made to feel dependent on the country they live in, we did not find evidence that the association between system justification and environmental intentions depended on whether participants read a system-preservation or control message, but the likelihood of signing petitions did. Among participants assigned to a second control condition, who were not exposed to any message, there was a negative association between system justification and pro-environmental behaviour intentions, raising the possibility that both original study conditions attenuated this association. Our findings highlight both the importance of replication and the inclusion of a true control condition, and they raise the possibility that leveraging an audience’s existing values may not always mobilise pro-environmental actions. In the case of ideological opposition to the status quo, a system dependence message could depress otherwise high pro-environmental responses.

## Introduction

People higher in system justification – the tendency to view the system as legitimate, fair, and worthy of protection (Kay & Jost 2003) – are more likely to deny environmental problems and abstain from pro-environmental behaviours (Feygina et al. 2010). Feygina et al. (2010) suggested this association occurs because acknowledging climate change entails an admission the system is perhaps *not* legitimate or fair, and because acting to resolve the issue demands a change to the system. Based on this rationale, they developed a message framing pro-environmental change as preserving (rather than threatening) current cultural and economic practices. They reported that ‘it is possible to eliminate the negative effect of system justification on environmentalism by encouraging people to regard pro-environmental change as patriotic and consistent with protecting the status quo’ (p. 326).

This important finding invited a novel way of communicating about climate change *without* causing backlash or apathy and has influenced theory development on the foundations of conservative climate change denial (e.g., Clarke et al. 2019; Hennes et al. 2016; Jylhä & Akrami 2015). After more than a decade since the original study, the United States remains among the highest greenhouse-gas emitters (Ritchie 2019). Thus, system justification could theoretically still be a factor to be considered when framing climate messages in this country (for discussion regarding the relevance of cultural context, see Wullenkord 2022). Currently, evidence about system-sanctioned change framing comes from Feygina et al.’s (2010, Study 3) single study with a sample that is small by today’s standards (*N* = 41), and a recent global mega-study that differed in design and sampling approach (Vlasceanu et al. 2024). We aimed to conduct a close replication of Feygina et al.’s seminal research.

### System Justification as a Barrier to Pro-Environmentalism

System justification theory (SJT; Jost & Banaji 1994) provides one answer for why we endure existing social, economic, and political arrangements that disproportionately disadvantage some, *even if* we are the ones facing the disadvantage (Jost et al. 2004). System justification is a motivation to rationalise and justify the existing system as fair, good, and inevitable. People with system justification tendencies may actively defend the status quo by, for example, opposing redistributive policy and accepting stereotypes that reinforce inequality (Jost et al. 2004). System justification therefore dissuades people from taking action that challenges the status quo, even if doing so would improve it to their benefit.

Despite its consequences, engaging in system justification is theorised to reduce the anxiety and uncertainty of current unequal sociopolitical systems (Jost & Hunyady 2002). Jost and Hunyady (2002) summarise that system justification prevents stress by giving the impression of a stable and just society; serves as a coping resource by making people feel hopeful and in control; and as a coping response when exposed to threat (thus, threats may bolster this defensive motivation). Putting these functions in the context of climate change, system justification may entail denial of the problem or acceptance of unconstructive hope, as examples.

Environmental problems can be perceived as a threat to the system in several ways. Feygina et al. (2010) argue that being motivated to see the current economic, political, and social systems as legitimate and beneficial prevents people from acknowledging the scale of impending environmental disaster. Accepting that these favoured systems have caused such destruction would challenge perceptions of legitimacy and fairness. They also noted the conflict between environmental protection and the current economic model; environmental solutions may unacceptably threaten the prevailing status quo that those engaging in system justification seek to defend. Supporting the theorised link between system justification and environmentalism, Feygina et al. (2010) showed that stronger endorsement of system justification corresponds with greater denial of environmental problems (Studies 1 and 2) and less frequent environmental behaviour (Study 2).

Building on Feygina et al.’s (2010) work, Campbell and Kay (2014) argued that the political opinion gap on climate change can be explained by solution aversion: a motivated denial of climate change stemming from the view that pro-climate reforms entail unacceptable threats. They showed that Republican climate denial is partly explained by the perception that climate solutions will adversely affect the American economy. Clarke et al. (2019) similarly found that the effects of right-wing ideological attitudes (in this case, right-wing authoritarianism and social dominance orientation) on climate denial were mediated by ‘mitigation threat’: the perception that climate policy threatens the social and economic status quo. Indeed, when environmental protection is framed as having negative implications for economic growth, there is a stronger ideological resistance (Harring & Sohlberg 2016). Ideologies that favour the current system, including system justification, may therefore be important barriers to pro-environmentalism.

### The Possibility of System-Sanctioned Change

If anti-environmentalism stems from a belief that acceptance of environmental problems (and their solutions) poses a threat to the system, then system-supportive reforms may be more palatable. This is the logic behind Feygina et al.’s (2010, Study 3) experimental research. Feygina et al.’s study began by reminding all participants (control condition and experimental condition) of their dependence on the country they live in. They did this using a paragraph designed to make participants’ relationship to the prevailing socioeconomic system salient. The text (see Table 1) stressed the importance of country of residence for participants’ life and wellbeing, including their financial wellbeing (e.g., via taxes they must pay, jobs available to them), social and personal wellbeing (e.g., quality of social services and access to leisure activities), and even their chances of finding happiness with a life partner. Participants then read an excerpt about research interest in environmentalism. After this, only the experimental (system-preservation) condition participants read the following additional text: ‘Being pro-environmental allows us to protect and preserve the American way of life. It is patriotic to conserve the country’s natural resources’. This short message avoided framing environmentalism as a challenge to the system, and instead positioned environmental action as a way to preserve the system. That is, it framed environmentalism in a way that works ‘with rather than against system justification motivation’ (Feygina et al. 2010, p. 333).

**Table 1:** Manipulation Text Presented to Each Condition.


	ORIGINAL STUDY CONTROL	SYSTEM-PRESERVATION EXPERIMENTAL	TRUE CONTROL

Salience of the system manipulation	WELL-BEING DEPENDS ON COUNTRY OF RESIDENCE Many people feel that the decision they make in terms of where to live is a very important one. In fact, recent surveys report that even at age 40, people still consider that their choice to live where they do was one of the most impactful decisions of their life. Indeed, sociological studies comparing the outcomes of residents of various countries show that there might be some truth to these perceptions. In particular, it seems that the country you live in has enormously broad effects on your life and well-being. In terms of financial well-being, for instance, the taxes you pay, the job and investment opportunities made available to you and the general state of the economy are all to a large extent under the control of your country’s government. But even in terms of social and personal well-being, the country you live in has substantial impacts: the quality of your social services (health and education), the leisure activities you have access to and time to pursue, even the likelihood that you will be happy with your eventual life-partner – all these aspects of your life are ones that are, at least according to these studies, to some degree dependent on the country you live in.		No text presented.

Environmental message	Researchers have always been interested in the state of the natural environment and have paid attention to how it has changed over the years. Today, researchers are especially interested in the relationship between people and the environment.		

		Being pro-environmental allows us to protect and preserve the American way of life. It is patriotic to conserve the country’s natural resources	


Although the manipulation was brief, Feygina et al. reported that it was effective. Specifically, they described significant interactions between system justification and messaging condition on pro-environmental intentions and the likelihood of signing a petition. System justification predicted lower behavioural intentions and lower likelihood of signing petitions in a control condition, and this association vanished for the system-preservation experimental condition.

### Justification for Replication

There is a growing attention to the importance of replication of research results. In general, framing studies in environmental communication are rarely replicated, and several recent replication failures (e.g., Kim et al. 2021, 2023; Stanley et al. 2021a) highlight that our field is not immune to replicability concerns. When considering the changes in environmental awareness and narratives in society, it would be crucial to not only develop new communication frames, but to also re-evaluate the effectiveness of the ones that have been developed in previous research.

The current empirical evidence for system-preservation framing is limited to Feygina et al. (2010, Study 3), which was conducted more than a decade ago and had some limitations, and a global mega-study (Vlasceanu et al. 2024). Feygina et al. (2010, Study 3) used a small (*N* = 41, 73% women) student sample from New York University (NYU), spread across two conditions. They described finding a significant system justification – experimental condition interaction for both pro-environmental behaviour intentions and signing environmental petitions. However, these key findings were interactions with a continuous variable (system justification), suggesting the impact of the framing depends on the level of system justification, and there is no detail about the distribution of responses on this scale. Mean levels of system justification tend to be low (Vargas-Salfate et al. 2018). While Feygina et al. (2010) did not report descriptive statistics for system justification in their study, Cichocka and Jost (2014) published a review of mean levels of system justification that includes data collected at a similar time using NYU students. In all cases, mean scores are below the scale midpoint of the same system justification measure.^1^

To interpret the significant overall interaction effect on intentions to engage in pro-environmental behaviours, Feygina et al. (2010, Study 3) reported results from two follow-up tests, which we summarise in Table 2. They examined those high and low in system justification (defined by *M* ± 1*SD* mean), reporting higher pro-environmental intentions for high system justifiers in the experimental condition: *p =* .09. This *p*-value is above the conventional alpha level, and a *p*-value reported as *p* > .10 was taken as evidence that low system justifiers did *not* respond differently to the messages. They also examined the relationship between system justification and pro-environmental intentions, separately by condition. They reported a negative association between system justification and pro-environmental intentions in the control condition (reported as ‘marginal’, *p* < .09), which ‘was eliminated’ (p. 334) in the system-preservation condition (*p* = .14).

**Table 2:** Summary of Results and Interpretation in Feygina et al. (2010, Study 3) and Our close replication Based on Only the Original Study Conditions (Original Study Control vs. System-Preservation Condition).


	FEYGINA ET AL. (2010, STUDY 3)		OUR CLOSE REPLICATION	

**Pro-environmental intentions**	Interpretation	Results reported	Interpretation	Results

Main effect of system justification	‘marginal’ … ‘in the expected direction’	*b* = 0.46, *SE* = 0.26, β = .36, *t*(37) = 1.75, *p* = .09.	No	*b* = 0.05, *SE* = 0.09, β = .04, *t*(374) = 0.59, *p* = .553

Main effect of condition	No	*b* = 0.20, *SE* = 0.52, β = .06, ns	No	*b* = –0.40, *SE* = 0.50, β = –.11, *t*(374) = –0.81, *p* = .421

Interaction between system justification and condition	Yes	*b* = 0.95, *SE* = 0.41, β = .49, *t*(37) = 2.29, *p* = .03	No	*b* = 0.05, *SE* = 0.12, β = .06, *t*(374) = 0.43, *p* = .670

**Follow-up test 1 – comparing high (*M* + 1*SD*) versus low (*M* – 1*SD*) system justifiers**				

Difference between conditions for high system justifiers?	Yes; PEB intentions were more pro-environmental in the system-preservation condition than the control	*b* = 1.40, *SE* = 0.82, β = .44, *t*(37) = 1.72, *p* = .09	N/A – follow up tests not performed given non-significant interaction	

Difference between conditions for low system justifiers?	No; PEB intentions were not reliably different across conditions.	*b* = –1.00, *SE* = 0.65, β = –.32, *t*(37) = –1.53, *p* > .10		

**Follow-up test 2 – examining relationship between system justification and PEB intentions in each condition**				

Control condition	‘A negative (albeit marginal) relationship’	*b* = –0.46, *SE* = 0.26, β = –.36, *t*(37) = 1.75, *p* < .09	N/A – follow up tests not performed given non-significant interaction	

Experimental condition	‘negative relationship was eliminated’ … ‘no reliable relationship’	*b* = 0.49, *SE* = 0.32, β = .39, *t*(37) = 1.53, *p* = .14		

**Petitions**	Interpretation	Results reported	Interpretation	Results

Main effect of system justification	‘marginal’, indicating system justification was inversely related to signing petitions	*b* = –0.64, *SE* = 0.38, Wald = 2.84, *p* = .09	Yes	*b* = –.35, *SE* = 0.10, 95% CI [–0.54, –0.16], Odds ratio (OR) = 0.70, Wald = 13.08, *p* < .001

Main effect of condition	No	*b* = 0.58, *SE* = 0.65, Wald = .82, ns	Yes	*b* = –1.36, *SE* = 0.54, 95% CI [–2.43, –0.31], OR = 0.26, Wald = 6.37, *p* = .012

Interaction between system justification and condition	Yes	*b* = 1.26, *SE* = 0.57, Wald = 5.01, *p* = .03	Yes	*b* = 0.37, *SE* = 0.13, 95% CI [0.12, 0.63], OR = 1.45, Wald = 8.20, *p* = .004

**Follow-up test 1 – comparing high (*M* + 1*SD*) versus low (*M* – 1*SD*) system justifiers**				

Difference between conditions for high system justifiers?	No	None; inferred from statement ‘reframing environmentalism as supporting (rather than undermining) the American way or life eliminates the negative effect of system justification on pro-environmental behavior’. Graph shows cross-over interaction.	Yes. But note that robustness checks described in text indicate no significant difference at *M* + 1*SD* system justification; differences emerge at extremely high levels of system justification in exploratory analyses.	49.33% of high system justifiers (*M* + 1*SD*) sign most petitions in the system-preservation condition versus 34.40% in the control condition.
	
Difference between conditions for low system justifiers?	Yes		Possible backfire? But note that robustness checks indicate no significant difference at *M* – 1*SD*, or *any* low level of system justification.	60.59% of low system justifiers (*M* – 1*SD*) sign most petitions in the original study control condition versus 47.48% in the system-preservation condition.

**Follow-up test 2 – examining relationship between system justification and petitions signed in each condition**				

Control condition	‘marginal negative effect of system justification on the probability of signing petitions’	None	Negative effect of system justification on the likelihood of signing petitions	*b* = –0.35, *SE* = 0.10, 95% Asymptotic CI [–0.54, –0.16], Wald = 13.08, *p* < .001

Experimental condition	‘no longer a reliable relationship’	*b* = 0.63, *SE* = 0.41, Wald = 2.33, ns	No effect	*b* = 0.02, *SE* = 0.09, 95% Asymptotic CI [–0.15, 0.20], Wald = 0.08, *p* = .783


To follow up the significant condition × system justification interaction on the likelihood of signing pro-environmental petitions, Feygina et al. (2010, Study 3) described a marginal negative relationship between system justification and likelihood of signing petitions in the control condition, and no relationship between these variables in the system-preservation condition. Statistics were not completely reported, though a figure showing the likelihood of signing petitions at high (*M* + 1*SD*) and low (*M* – 1*SD*) levels of system justification implies a greater likelihood of signing petitions for high system justifiers in the system-preservation condition than in the control condition. Besides the interaction itself (where *p* = .03), no *p*-values were reported for the analyses on the likelihood of signing petitions. Therefore, while Feygina and colleagues (2010, Study 3) developed an interesting and well-reasoned theoretical account of the system justification-environmentalism association that has had a profound impact on the field, the evidence they presented does not yet make a compelling case for system-preservation framing. An intervention based on system-preservation framing was recently included in Vlasceanu et al.’s (2024) 63-country study examining the effects of 11 pro-environmental interventions. Their intervention emphasised participants’ dependence on where they live, gave examples of the ways in which climate change is threatening this way of life, and invited pro-environmental action to protect and preserve their way of life. The intervention showed small effects on belief in climate change, policy support, and intentions to share climate information on social media by (each increased by 0.85%, 0.74%, and 5.81%, respectively), and did not affect effort in a tree planting activity. Reanalysis of these data across political liberals and conservatives indicated that the intervention increased climate beliefs of both groups, increased climate policy support only among political liberals, and backfired to *decrease* tree planting efforts among political conservatives (Berkebile-Weinberg et al. 2024). These mixed results for interactions with political orientation further warrant a closer replication of Feygina et al.’s study that directly measures system justification.

### Current Study

We conducted a close replication of Feygina et al. (2010, Study 3) using a larger, general U.S. population sample. We tested the effects of system justification across conditions (control, system-preservation condition) on intentions to act pro-environmentally and on agreement to sign pro-environmental petitions. This was not a direct replication. Deviations from the original study protocol are summarised in Table 3 and included a shift to online recruitment (the original experiment was completed in a controlled lab setting) and implementing strategies to ensure the quality of data collected in this setting (i.e., attention and naivety checks). Given that we were conducting the replication more than ten years on from the original, the petitions were also updated to reflect environmental issues still relevant today.

**Table 3:** Overview of Study Differences between Original Study and Our Close Replication.


	FEYGINA ET AL. (2010, STUDY 3)	OUR CLOSE REPLICATION

**Sample**	41 New York University undergraduate students (30 women, 11 men). No age or nationality information reported.	567 adults living in, and identifying their nationality as, United States of America (276 women, 272 men). Ages ranged from 18 to 93 years (*M* = 37.06, *SD* = 13.92).

**Setting**	Computers in a controlled, in-person laboratory setting.	Online study; participants took part in their own environment.

**Conditions**	1) Original control condition, 2) System-preservation experimental condition.	1) Original control condition, 2) System-preservation experimental condition, 3) True control condition.

**PEB intentions**	Identical; pro-environmental behaviour intentions (10 items, 9-point response scale). Analysis used the mean score of 10 items as the dependent variable in a linear regression analysis.	

**Petitions**	Seven pro-environmental petitions presented after a false debrief, ostensibly unrelated to the study. Created a 3-point measure by recoding data into: *no petitions signed* (34.1% of participants), *a few petitions signed* (i.e., one to three petitions, 29.3%), *most petitions signed* (i.e., four to seven petitions, 36.6%), to use in an ordered logistic regression.	Seven pro-environmental petitions presented online and before the debrief, updated for relevance. Created a 3-point measure by recoding data into: *no petitions signed* (34.0% of participants), *a few petitions signed* (i.e., one to three petitions, 16.9%), *most petitions signed* (i.e., four to seven petitions, 49.0%), to use in an ordered logistic regression. In addition, analysed data using the count of how many petitions were signed, using a negative binomial regression.


A successful replication of Feygina et al.’s (2010, Study 3) conclusions ought to show a significant interaction between system justification and experimental condition on pro-environmental behavioural intentions. Probing this interaction using the same two methods as the original study should show that for participants high in system justification (defined by Feygina et al. as scoring at *M* + 1*SD*), behavioural intentions are significantly higher among participants assigned to the system-preservation condition than the control condition. For those with low levels of system justification (*M* – 1*SD*), there should be no significant differences in responses across conditions. This pattern should also be reflected in the simple slopes analysis: system justification ought to be significantly, negatively related with pro-environmental behavioural intentions in the control condition, and unrelated in the system-preservation condition. This would provide evidence that the system-preservation frame successfully eliminated the negative effect of system justification on environmental intentions.

As an extension to the replication attempt, we also introduced a second control condition, which we refer to as the ‘true control’ condition. In it, participants received no message or information invoking salience of the system. Jost et al. (2004) explain that people ‘want to hold favourable attitudes about social and political systems that affect them’ (p. 887). The initial text Feygina et al. (2010, Study 3) showed all participants was designed to emphasise one’s reliance on the system and may have affected system justification tendencies to shape responses in the experiment. Specifically, people who feel more dependent on the system should be more motivated to defend it (Feygina et al. 2010), and in turn, respond less pro-environmentally. A similar ‘system dependence’ message was used in Hennes et al. (2016) to manipulate system justification, with effects on the accuracy of recall of information of climate change. Thus, the salience of the system manipulation could have depressed pro-environmental responding in both original study conditions, meaning that the positive results in the system-preservation condition could reflect a *restoration of typical* pro-environmental responding, rather than a true increase relative to baseline. We test this possibility by comparing responses within the original study conditions to our true control condition. Finally, if the system-preservation framing successfully engages political conservatives, then the patterns described above ought to replicate with political orientation in place of system justification.

## Method

The University human research ethics committee approved ethical aspects of this study (#2022/304). The pre-registration of this research, study materials, data, and R code, are available on the OSF: https://osf.io/7xpr6.

### Participants

Feygina et al.’s (2010, Study 3) sample was 41 New York University students (73% women). We simulated power at a critical *p*-value = .05 in a two-tailed test using the *paramtest* package (Hughes 2017) in R (R Core Team, 2022), based on the effect sizes reported in the original study for pro-environmental behavioural intentions (*R^2^* = .10, *b1* = –0.36, *b2* = 0.06, *b3* = 0.49; see https://osf.io/6b8kz for the R script and https://osf.io/q7wr9 for the output). A final sample of 170 would secure 90% power for this interaction effect. Although our goals involving the true control condition were exploratory, we expected that the salience of system manipulation could heighten the negative association between system justification and pro-environmentalism in the original study control condition and amplify the positive moderating effects of system-preservation framing between system justification and pro-environmentalism (see Diamond 2020). Based on these expectations, we simulated a graph and conducted the power analysis (see https://osf.io/q7wr9), *R^2^* = .10, *b1* = –0.25, *b2* = –0.20, *b3* = –0.15, *b4* = –0.35, *b5* = 0.50; see https://osf.io/295wt for the R script and https://osf.io/q7wr9 for the output and simulated graph). To attain 90% power to detect significant interaction effects in the analysis including all the three conditions, our minimum sample size was 480. We increased this figure by 20% (to *N* = 576) to ensure sufficient power after applying our preregistered exclusion criteria.

Between 25–26th August 2022, we recruited participants living in, and identifying their nationality as, the United States of America using Prolific’s balanced sample feature to recruit men and women equally. Participants received 0.97GBP for completing the 7-minute Qualtrics survey.^2^ We elected to recruit participants via Prolific because comparisons to other platforms suggest the data collected through Prolific are of higher quality (Peer et al. 2021). We advertised the survey using the title ‘Evaluating the clarity of news articles’ to align with the cover story presented in the original experiment, with the Participant Information Sheet claiming that the project explores ‘how the language used in newspaper articles affects how clear and interesting this information is to readers’. Following our preregistered exclusion criteria, we removed three participants who withdrew consent and six who did not identify their nationality as the U.S. We report analyses with the remaining 567 participants,^3^ ranging in age from 18 to 93 years (*M* = 37.06, *SD* = 13.92; 48.68% women, 47.97% men, 3.35% non-binary or prefer not to say) and predominantly identifying their race as White (76.37%), followed by Asian (9.00%), Black or African American (6.88%), Some other race (4.23%), Prefer not to say (1.76%), American Indian or Alaska Native (1.24%), and Native Hawaiian or Other Pacific Islander (0.53%). The sample leaned liberal (*M* = 34.62, *SD* = 27.85 on a 0–100 liberal-conservative scale).

## Materials and Procedure

Participants were randomly assigned to one of the three conditions. All material was presented in the same order as in the original lab-based study, except for the petitions. Our Qualtrics file and a survey printout are on the OSF page.

### Salience of the System Manipulation

Participants in the original study conditions (system-preservation condition, control condition) read instructions obscuring the aims of the study (‘First, please read this excerpt from a newspaper article. Later, you will be asked some questions about it, so please read it carefully.’). Next, a paragraph noted participant’s dependence on the country they live in to make their relationship to the prevailing socioeconomic system salient (see Table 1).

### Environmental Passage and Manipulation

Original study participants read: ‘Researchers have always been interested in the state of the natural environment, and have paid attention to how it has changed over the years. Today, researchers are especially interested in the relationship between people and the environment.’ In the system-preservation condition, participants additionally read: ‘Being pro-environmental allows us to protect and preserve the American way of life. It is patriotic to conserve the country’s natural resources.’ To bolster the cover story, the participants then rated in both conditions how clear, easy to understand, interesting, and persuasive, these passages were. ‘True control’ condition participants began the experiment with the questions below.

### Pro-Environmental Behaviour Intentions and System Justification

As in the original study, we used the 10-item scale that measures intentions to engage in pro-environmental behaviour (α = .93, item example: ‘I intend to join and provide financial support to pro-environmental organisations in the near future’), and the 8-item system justification scale (α = .88, item example: ‘Everyone has a fair shot at wealth and happiness’, Kay & Jost 2003), each rated from 1 (‘strongly disagree’) to 9 (‘strongly agree’). Feygina et al. (2010) reasoned that system justification ought to be measured towards the end of the experiment to further obscure the study aims and reported no difference in system justification by condition. Our results are consistent with all three conditions compared: *F*(2, 564) = 0.65, *p* = .521, η^2^ = .002.

### Pro-Environmental Petitions

Feygina et al. (2010) concluded their study using a false debrief, then presented participants with seven pro-environmental petitions ostensibly arranged by an on-campus environmental group and unrelated to the study. Due to the difficulty of displaying an onscreen false debrief followed by petitions that are believably unlinked to the study, our method deviated here: instead, we updated the original petitions by removing outdated references (see https://osf.io/gbfnh) and asked participants whether they would like to sign each petition (yes/no), with (false) information that they would receive links to their chosen petitions at the end of the survey. Following Feygina et al. (2010), we recoded responses into ordered categories (see Table 2).

### Survey End

We recorded age, gender, political orientation, and subjective social status using the MacArthur Scale (Adler et al. 2000). We also asked two questions to verify participants met the eligibility criteria for the study (located in, and identifying their nationality as, the United States). The in-person nature of the original experiment gave the researchers more experimental control and oversight of participants. To ensure a similar level of data quality in the online, anonymous setting, we captured time spent on the survey and included a multiple-choice attention check question, and two yes/no closed-ended naivety check questions based on Kim et al. (2021), detailed in the Supplementary Materials. Finally, we disclosed the true study aims and gave participants the option to withdraw from the study in line with our ethics protocol.

## Results

We used R (R Core Team 2023) for the following analyses (see the R scripts and outputs on the OSF). We used the *emmeans* package (Lenth 2023) to conduct simple slope analyses, the *MASS* package (version 7.3–58.4; Venables & Ripley 2002) to conduct ordered logistic and negative binomial regression analyses, and the *Dunnet* method to adjust for multiple comparisons for simple slope analyses that include the true control condition. The *ufs* package (Peters & Gruijters 2021) estimated scale reliability. The *ggplot2* package (Wickham 2016) and the *interactions* package (Long 2021) helped us visualise the interactions and compute Johnson-Neyman intervals, respectively.

### Descriptive Statistics of Outcome Variables in Each Condition

Table 4 presents descriptive statistics for PEB intentions and total number of pro-environmental petitions signed across each experimental condition. It shows that on average, participants scored just below the scale midpoint in their intentions to engage in pro-environmental behaviour, indicating slight disagreement that they will take the actions specified in the measure. Of the seven pro-environmental petitions they were presented, participants on average agreed to sign just over three.

**Table 4:** Means (standard deviations) for Pro-Environmental Variables for Each Experimental Condition.


	PRO-ENVIRONMENTAL BEHAVIOUR INTENTIONS	TOTAL PETITIONS SIGNED

System-preservation condition (*n* = 190)	4.48 (1.93)	3.17 (2.76)

Original study control condition (*n* = 188)	4.67 (1.72)	3.28 (2.95)

True control condition (*n* = 189)	4.59 (1.87)	3.31 (2.88)


### Close Replication: Effects in the Original Study Control Condition vs System-Preservation Condition

We present results from analyses that aimed to replicate those reported in Feygina et al. (2010, Study 3) alongside the original study findings in Table 2. In contrast to our predictions and Feygina and colleague’s findings, the relationship between system justification and PEB intentions did not depend on messaging condition in our study. We appeared to replicate the significant interaction between system justification and condition on likelihood of signing petitions. Table 2 shows that simple slope analyses identified a significant negative effect of system justification on likelihood of signing petitions in the original study control condition, and no effect in the system-preservation condition. This finding aligns with the pattern of results described by Feygina et al. (2010, Study 3) and indicates that in the absence of a system-preservation frame, a stronger tendency to justify the system predicts a lower likelihood of signing environmental petitions; an effect that is non-significant with the frame.

Proceeding with robustness checks, we found that the significant interaction between system justification and condition on the number of petitions signed remained even when repeating these analyses using the actual count of petitions signed, using negative binomial regression (*b* = 0.16, *SE* = 0.08, *p* = .035).^4^ There was a significant, negative relationship between system justification and petitions signed in the original study control condition (*b* = –0.53, *SE* = 0.18, 95% Asymptotic CI [–0.88, –0.18], *p* = .003), and no significant relationship in the system-preservation condition (*b* = –0.01, *SE* = 0.17, 95% Asymptotic CI [–0.35, 0.32], *p* = .930).

The difference in the number of petitions signed across conditions was not found for participants at *M* – 1*SD* (2.23) and *M* + 1*SD* (5.30) on system justification. However, we used exploratory Johnson-Neyman analyses to examine at what level of system justification participants responded differently depending on the condition. This analysis identified a significant effect of condition (original study control versus system-preservation) on the number of petitions signed among those with extremely high levels of system justification (i.e., values above 7.48 on the scale, which ranged from 1 to 9). This result ought to be interpreted with caution, because it is based on a fitted model, and individuals with extremely high values on system justification are rare.

### Extension to the Replication: Analyses with a True Control Condition

#### Pro-Environmental Behaviour Intentions Across Three Conditions

**Figure 1:**
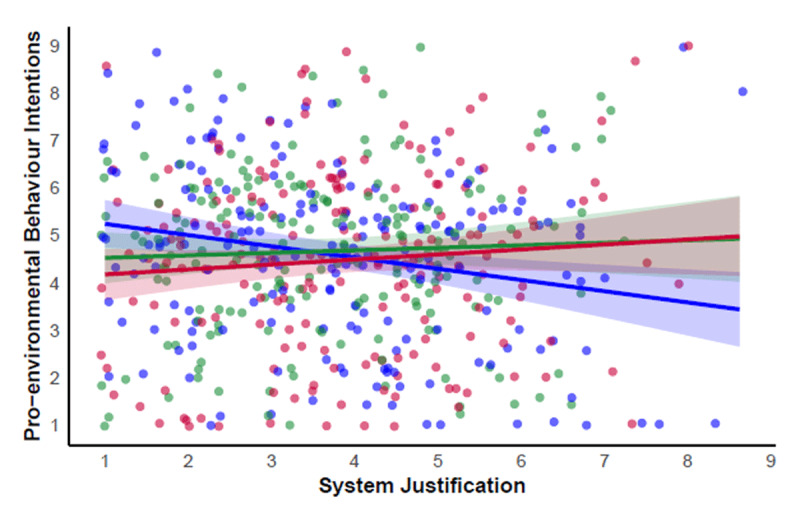
Associations between System Justification and Pro-environmental Behaviour Intentions in the System-Preservation Condition (Red), Original Study Control Condition (Green) and True Control Condition (Blue). *Note*. Shaded areas represent 95% confidence intervals. Data points are jittered to avoid overlap.

We then examined PEB intentions across all three conditions, setting true control condition as a reference group. This analysis returned two significant interactions: the first suggests the effect of system justification on PEB intentions depends on whether participants are in the original or true control condition (*b* = 0.29, *p* = .015), and the second suggests the effect on PEB intentions depends on whether participants are in the system-preservation experimental condition or true control condition (*b* = 0.34, *p* = .003). Analysis of simple slopes showed a significant negative relationship between system justification and PEB intentions only in the true control condition (*b* = –0.24, *SE* = .08, *p* = .003). The association was non-significant in both the original study control condition (*b* = 0.05, *SE* = .09, *p* = .553) and system-preservation condition (*b* = 0.11, *SE* = .09, *p* = .213), raising the intriguing possibility that *both* original study conditions eliminated the negative effect of system justification on PEB intentions (see Figure 1).

Comparing PEB intentions of those with lower system justification (*M* – 1*SD*, 2.18) highlights significantly *lower* PEB intentions in the system-preservation condition (*M* = 4.30, *SE* = 0.20) than the true control condition (*M* = 4.97, *SE* = 0.18), *t*(561) = –2.49, *p* = .025. The system-preservation message therefore lowered the PEB intentions of those rejecting system justification. For those higher in system justification (*M* + 1*SD*, 5.37), PEB intentions were not significantly higher in the system-preservation condition (*M* = 4.64, *SE* = 0.18) than in the true control condition (*M* = 4.21, *SE* = 0.18), *t*(561) = 1.65, *p* = .179. Although these comparisons at *M* ± 1*SD* align with Feygina et al.’s (2010, Study 3) analytic approach, the values are somewhat arbitrary for probing the interaction. We therefore conducted an exploratory Johnson-Neyman analysis to identify at what level(s) of system justification there is a significant effect of condition on PEB intentions. This indicated that the effect of the system-preservation framing versus the true control condition on PEB intentions was statistically significant both at levels of system justification below 2.87 and above 5.83 on the 1–9 Likert-type scale. Thus, there was some evidence that participants higher in system justification responded differently depending on condition. As shown in Figure 1, the true control condition is a context where PEB intentions appear lowest for high system justifiers, and highest for low system justifiers.

#### Petitions Signed across Three Conditions

The effect of system justification on signing none, a few, or most petitions did not differ between original and true control conditions (*b* = –0.09, *SE* = 0.13, 95% CI [–0.34, 0.16], OR = 0.92, *p* = .498). However, it differed between true control and system-preservation conditions *(b* = 0.28, *SE* = .12, 95% CI [0.04, 0.52], OR = 1.33, *p* = .021). Simple slope analysis revealed significant negative relationships between system justification and petition signing in the original (*b* = –0.35, *p* < .001) and true control conditions (*b* = –0.26, *p* = .002), and non-significant association in the system-preservation condition (*b* = 0.02, *p* = .786). At 1*SD* below the mean, lower system justifiers were more likely to indicate willingness to sign most petitions in the true control condition (60.68%) or original control condition (61.36%), compared to the system-preservation condition (48.01%). For higher system justifiers, the highest percentage were willing to sign most petitions in the system-preservation condition (49.90%) compared to the true control condition participants (40.39%) and original study control condition (34.58%; see Table 5 for full results and percentages at *M* ± 1*SD* and *M* ± 1.5*SD*).

**Table 5:** Percentages of Those Lower and Higher in System Justification Signing None, a Few, and Most Pro-Environmental Petitions in All Study Conditions.


	LOWER SYSTEM JUSTIFICATION		HIGHER SYSTEM JUSTIFICATION	

	*M* – 1.5*SD*	*M* – 1*SD*	*M* + 1*SD*	*M* + 1.5*SD*

**Original study control condition**				

None	18.80	23.36	47.79	54.65

A few	13.56	15.28	17.62	16.70

Most	67.65	61.36	34.58	28.65

**System-preservation condition**				

None	34.82	34.39	32.70	32.29

A few	17.65	17.60	17.40	17.34

Most	47.53	48.01	49.90	50.37

**True control condition**				

None	20.34	23.87	41.67	46.74

A few	14.20	15.45	17.94	17.71

Most	65.47	60.68	40.39	35.55


**Figure 2:**
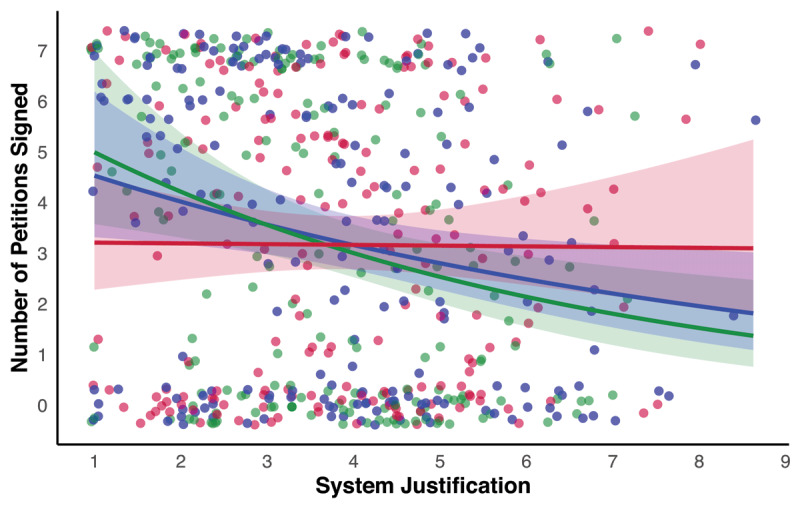
Associations between System Justification and the number of pro-environmental petitions signed in the System-Preservation Condition (Red), Original Study Control Condition (Green) and True Control Condition (Blue). *Note*. Shaded areas represent 95% confidence intervals. Data points are jittered to avoid overlap.

However, our robustness checks that involved negative binomial regression using the count data identified no significant interaction between system justification and true control versus original study control condition (*b* = –0.05, *SE* = 0.08, *p* = .516) or between system justification and true control versus system-preservation condition (*b* = 0.12, *SE* = 0.07, *p* = .114). Thus, there was not robust evidence that system-preservation framing enhanced the likelihood of individuals with higher levels of system justification signing pro-environmental petitions relative to our true control condition (see also Figure 2).

### Effects with Conservative Political Orientation

Political orientation did not interact with conditions to predict either outcome variable (see Supplementary Materials for full details).

## Discussion

Feygina et al.’s (2010) findings highlighted the potential to reinvigorate conservative engagement in environmental movements with a simple intervention: framing environmentalism as key to preserving the American way of life. Our close replication of this important finding returned mixed evidence about system-preservation framing. We did not find evidence that the effect of system justification on pro-environmental intentions depends on exposure to this system-preservation message when compared to the original study control condition. Instead, system justification did not predict PEB intentions. While this null finding may call into question whether system justification is an ideology with robust negative implications for environmental outcomes, we found a negative effect of system justification in our true control condition, which is discussed later in this manuscript.

A significant interaction between conditions and system justification on petition signing also showed that in the original study control condition, system justification predicted lower likelihood of signing pro-environmental petitions, while it was not related to petition signing for participants exposed to the system-preservation message. This pattern does replicate Feygina et al.’s (2010) work. However, in the system-preservation condition, the apparent increase in the likelihood of signing ‘most petitions’ for those higher in system justification was matched by a decrease for those lower in system justification. With different treatment of the data that retains variance in the number of petitions signed, we replicated the interaction and locate differences in responses only at extremely high levels of system justification. Taken together, our findings therefore do not provide strong support for the idea that framing environmentalism as a case of system-sanctioned change motivates people to help the environment, and they raise questions about whether such messaging backfires, and about the extent to which the dependence on the system manipulation shaped responses in the original study conditions.

### Potential Explanations for the Replication Failure

The move to online data collection was necessary to secure a large sample, however it offered less experimenter oversight than the original experiment. It also prevented us from offering petitions ostensibly unrelated to the study. The way Feygina et al. (2010, Study 3) covertly measured petition signing was a key strength of their research that we were not able to incorporate into the design of our close replication, which could have affected results. Our participants were likely more aware that petition signing was a dependent variable of interest, potentially leading them to (dis)agree to sign at different rates. Social psychological research is increasingly moving online (Sassenberg & Ditrich 2019). Clifford and Jerit (2014) suggest that participants might respond differently to lab- and online-based studies on a few domains, such as looking up answers in knowledge questions, though attentiveness and socially desirable responding does not appear to differ. Nevertheless, in-person replication attempts of the system-preservation frame are needed to rule out the possibility that the move online affected responses and as a potential explanation for our findings.

It is also important to note that our participants did not give responses consistent with a motivation ‘to defend, bolster, and justify the social, economic, and political systems on which they depend’ (Jost et al. 2017, p. 101). Mean scores on the 1–9 system justification scale were below the theoretical scale midpoint (i.e., *M* = 3.77, *SD* = 1.60, scale midpoint = 5). As a result, at ‘high system justification’ (*M* + 1*SD*), participants endorsed the items only weakly (i.e., scoring 5.37). Vargas-Salfate et al.’s (2018) 19-nation investigation of system justification found that means are typically low; on a 1–7 scale, the mean in their U.S. sample was 3.41, and levels ranged from 2.43 (in Brazil) to 4.21 (in China: the only nation with mean scores above the theoretical scale midpoint). Feygina et al. (2010) did not report mean system justification in their sample, though given the overall sample size and recruitment from NYU, we could speculate they would have had few participants nearing the top end of the scale. Examining mean system justification tendencies among NYU undergraduates between 2007–2011 reported in Cichocka and Jost (2014) further reinforces concerns that our sample overall scored lower in system justification than Feygina et al.’s sample (i.e., reported means range from 4.41 to 4.53; see our Note 1). While we cannot exclude the possibility that system-preservation framing effectively motivates those strongly endorsing system justification, which could be tested through recruitment of more conservative samples in future, nevertheless the exploratory Johnson-Neyman analyses examined responses across the full range of system justification in our sample. These analyses found significant differences in PEB intentions among those high in system-justification (above 5.83) only when contrasting the system-preservation with the true control conditions, not with the original study control condition; and differences in petitions signing only at extremely high levels of system justification (above 7.48) when contrasting the system-preservation with the original study control conditions.

Another difference between our replication and the original research is the temporal context. The U.S. in August 2022, when our data were collected, was not a stagnant reflection of the U.S. pre-2010, when Feygina et al. (2010, Study 3) were preparing their manuscript. Indeed, the changing context is one potential explanation for why Baldwin and Lammers’ (2016) findings that conservatives respond more pro-environmentally to past-focused than future-focused messages has failed to replicate (Kim et al. 2021; Stanley et al. 2021a). Given the existing polarisation and abundance of environmental information, participants may have set views before entering the experiment (Bernauer & McGrath 2016), and the brief system-preservation message might no longer resonate with American conservatives or be powerful enough to shift responses in the pro-environmental direction. We call for more research on this topic that also considers plausible updates in the research design (see Stroebe & Strack 2014), including whether system justifiers would respond to a different message in today’s context. At the same time, the manipulation interacted with system justification and not with another ideological variable (political orientation), suggesting it was appropriately targeted to system justification.

Sotola and Credé (2022) recently examined experimental research on system justification to estimate threats to replicability, such as low power, publication bias, and questionable research practices. Their analysis indicated that given its low power, main effects within the SJT literature have an estimated replicability rate of just 14%, and that false positives may make up an estimated 60% of reported effects; for interaction effects, the estimated replicability rate is 19%, with a predicted false discovery rate of 25%. Interestingly, the system dependence manipulation (which was included, but not tested, in Feygina et al. 2010, Study 3) had the highest expected replication rate (67%) and lowest estimated false discovery rate (.03). Sotola and Credé conclude that overall, the replicability of SJT research is ‘likely to be low’ (p. 906).

### The Effects of System Justification in the Absence of Manipulation

The inclusion of our true control condition revealed that participants who were low in system justification reported higher intentions to engage in PEB when they were presented with *no* message than if they saw the system-preservation message and/or the system dependence message. Thus, seeing no information was better for maintaining intentions to engage in environmentally protective behaviours than presenting information aimed at changing people’s responses. There are a number of possible explanations for this backfire effect, but as it occurred in both original study conditions, one possibility is that the initial message designed to make their reliance on the system salient caused low system justifiers to respond more similarly to higher system justifiers.

Hennes et al. (2016, Study 3) reported on a system justification experiment that showed this is possible. In a control group exposed to no information, endorsement of system justification predicted poorer recall of climate change information, indicating lower acceptance of the seriousness of climate change. In the experimental group that was told about a U.S. recession, there was no main effect of system justification on recall, but an interaction effect was found showing reduced recall among those low in system justification (defined as *M* – 1*SD*), with no effect on those high in system justification (*M* + 1*SD*). Thus, when those who typically reject system justification motives were led to believe that the economy was under threat, they engaged in similar information processing as higher system justifiers to lessen the perceived severity of climate change. In other words, the manipulation made higher- and lower-system justifiers respond in the same way (and, interestingly, it did so without changing *levels* of system justification). Moreover, as further evidence, Kay et al. (2009, Studies 2 and 3) showed that the same system dependence manipulation that was used in Feygina et al. (2010) and in our replication leads to a greater deference to the current system one is motivated to justify.

Together, this literature supports the possibility that the system dependence manipulation engaged the situational system-serving needs of participants who otherwise tend to reject such motives. The result – that is, the flatter line we observe between system justification and pro-environmental behaviour intentions in both original study conditions – may be explained by system dependence affecting higher and lower system justifiers to respond in similar ways. Based on their reporting, it is unclear whether this effect was observed in Feygina et al.’s (2010) data or if it is unique to our replication effort. However, the effect could be specific to the pro-environmental intention outcome variable; for petition signing, both control groups (including the original study control, which viewed the system dependence message) responded similarly. Jost et al. (2012) argued that collective actions like petition signing likely reflect a venting of anger or attempt to persuade authority to change course. Thus, a possible explanation is that the system dependence message demotivated low system justifiers from making personal changes, though they still felt that signing pro-environmental petitions could alleviate their anger about environmental degradation in the original study control condition, unless they additionally read the system-preservation message that framed doing so as entrenching an undesirable status quo.

### Motivated to *Reject* the Status Quo

On average, participants in our sample tended *not* to endorse system justifying beliefs. We discussed this above, but here further consider that it lends a different interpretation of our results. Jost et al. (2011) stated that when system justification is low, people might engage in efforts to change the current system. Those who strongly reject the status quo may not simply lack a motivation to defend the system but feel compelled to actively seek change. What might be happening to depress low system justifiers’ pro-environmental intentions in the original study conditions in our sample? One explanation is that people who are open to changing and challenging the system (i.e., low in system justification) might have been less willing to do so after a reminder of the unchanging nature of the system and its influence on them. Osborne et al. (2018) previously warned that system-preservation messaging could backfire: ‘framing a progressive social movement as an extension of the status quo could ironically mobilize opposition among liberals, thus offsetting any potential gains in public support obtained by reaching out to conservatives’ (p. 15). When told that environmentalism will protect the system, those who reject the system may reject environmentalism. Interesting future directions are to omit the system dependence passage and test the system-preservation message alone and add a system-*challenging* message: framing environmental protection as a way to overthrow the status quo. We might expect this message to heighten low-system justifiers’ pro-environmental responses, including collective action.

Until recently, system justification motives have been studied as barriers to collective action. Jost et al. (2017) addressed this oversight by articulating a system-justification model of collective action (SJMCA). In it, they argued that system justification dampens perceptions of injustice and experiences of moral outrage. Anger is a key motivator of system-challenging protest (van Zomeren et al. 2008), including climate activism (Stanley et al. 2021b), so this model has implications for who is likely to engage in what sorts of collective action. They predicted that while activism against the prevailing system is more likely among those who are low in system justification, high system justifiers might take part in protest in defense of the system if it is under threat. One of the dependent variables studied here, petition signing, is a form of non-disruptive protest (Jost et al. 2012). Testing the effect of system-preservation framing within the context of the SJMCA could elucidate whether our unexpected effects are mediated by a reduction of eco-anger among low system justifiers caused by the system dependence manipulation, and thus a low motivation to take part in collective action.

In the absence of any manipulation (i.e., in our true control condition), we replicated the negative effect of system justification on environmental outcomes. Thus, efforts to experimentally manoeuver this association to increase environmentalism are still worthwhile. Jost et al.’s (2012, Study 3) research asked some teachers to detail which elements of the political, legal, social, or economic system they would *not* recommend other countries model on their nation. This manipulation reduced system justification and increased both anger directed at the government and willingness to protest. While learning of outsiders’ criticisms of the system is a common method to evoke threat that increases system justification motives (Kay & Friesen 2011, but see Sotola & Credé 2022), this finding suggests that generating ones’ *own* critiques could promote system challenging action. Given increasing public demands for ‘system change, not climate change’, a system-rejection task could be tested as a promising intervention in the climate domain.

### Alternative Explanations for Backfire Effects

Researchers use the terms ‘backfire effects’ (and sometimes, ‘boomerang effects’) to describe when climate messages have the opposite effect to that intended, such as increasing political polarisation on climate solutions (Hart & Nisbet 2011). This generally reflects a strengthening of right-wing opposition to climate action, though here we found some evidence of a depression of left-wing intentions to behave pro-environmentally. Swire-Thompson et al. (2020) has previously raised concerns about the replicability of backfire effects in the literature on misinformation corrections. They had several alternative explanations for these effects. For example, they suggested that the effect could be caused by measurement error given the misinformation literature is relatively new and that the majority of work that identified a backfire effect relied on single item measurement. Measurement of PEB is contentious and often uses ad hoc items or scales (Lange & Dewitte 2019), so this critique could apply to our research. Possible advances in measurement of PEB are discussed below.

Another possibility is that participants are responding based on their expectations of the research aims, and that the backfire is therefore caused by demand characteristics. Swire-Thompson and colleagues warn about this possibility because some research has found that ‘virtually all participants’ in a memory updating study correctly identified the study aims. Our two naivety checks identified 67 participants who either recalled taking part in a similar study before or having heard about research indicating that people who prefer the status quo are more persuaded by messages that describe environmental protection as preserving the American way of life. Our results did not change when these individuals were excluded. These and other alternative explanations, such as effects on emotions, ought to be investigated further in future research.

### Strengths, Limitations, and More Future Directions

Sotola and Credé (2022) called for researchers to adopt open science practices and for journals to encourage attempts to replicate SJT findings, both conceptually and directly. We contribute to this call by focusing on one of the most impactful SJT publications in our area. The open science practices, including preregistration and publicly available data, are strengths of our replication project. We also recruited a large sample to power examination of the interaction effects, which enabled us to look in an exploratory way across the range of system justification in our data. Inclusion of a true control condition allowed us to generate new insights about the effects of the original study conditions. Finally, including political orientation was an important advance that allowed us to examine the value of system-preservation framing for encouraging conservative environmentalism. However, all research programs have limitations, and the discussion above regarding potential explanations for the null findings identify several limitations of our work that warrant addressing in future replications.

Future research could also further interrogate the role of chronic individual differences in system justifying ideologies in peoples’ responses to climate change. Researchers are increasingly recognising that awareness of the reality of climate change is causing emotional challenges like eco-anxiety (e.g., Clayton 2020). Climate change is a profound global threat and may cause anxiety owing to uncertainty about how and when exactly we will be affected, and whether humankind will do enough to address the problem (Ojala et al. 2021; Pihkala 2020). As a motivation that addresses epistemic and existential needs to reduce both uncertainty and perceptions of threat (Jost et al. 2011), system justification tendencies could protect people from climate distress. Indeed, Jylhä (2016) found that self-reported tendency to avoid experiencing anxiety correlates with both system justification and climate change denial. However, Wullenkord et al. (2021) found no association between climate anxiety and system justification in cross-sectional research. If system justification processes buffer effects of climate threat on anxiety, experimental or longitudinal tests could be more appropriate to detect an association.

Further efforts to adequately measure behaviour are needed to understand the true effects of system justification on meaningful outcomes, and the success of interventions to curb these effects. The PEB intentions measure had some strengths, such as that it successfully avoided ceiling effects, and the modified petitions measure allowed us to deploy the study at scale. However, the intentions measure falls victim to the intention-action gap (i.e., we did not follow up to find out if people actually performed these behaviours), and the petitions measure may not have been believable or maintain the level of ecological validity with the cover story used in the original study. Lange and Dewitte (2019) reviewed alternative measures of behaviour to explore in future research, such as lab-based observation of a target behaviour or recording whether a participant elects to donate a portion of their participation incentive to an environmental cause. Some measures also require a personal sacrifice or effort on behalf of the participant, such as the Work for the Environment task (Lange & Dewitte 2022). Selecting appropriate measures for future research ought to involve considerations around which behaviours are relevant to system justification, such as forgoing high-emission traditions (e.g., abstaining from meat during celebrations where it is typically eaten).

Attitudes are another suitable target for this research to test theoretical predictions about the effects of system justification on rated support for policies and petitions for actions that variously challenge or protect the status quo. Some environmental policies have been criticised for being unjust for certain populations or too mild and ineffective, in part because politicians may aim to keep their supporters satisfied by proposing environmental solutions that maintain the status quo. Researchers could examine whether individuals high in system justification prefer tackling emissions through ‘techno-optimistic’ solutions that do not challenge the status quo (Marquardt & Nasiriyousi 2022), and conversely whether low system justification predicts greater support for policies and protest action that explicitly challenges the status quo through broader reform.

## Conclusions

Feygina and colleagues (2010) developed a well-reasoned theoretical account of the system justification-environmentalism association. Our replication attempt did not find a compelling case for system-preservation framing. However, our findings paint an interesting picture of potential ideological reactance of low-system justifiers to system-sanctioned change, and highlight that in the absence of manipulation, endorsement of system justification predicts less pro-environmental responses. We recommend further replication attempts to compare promising messages to a no-message control to fully comprehend their effects across ideological spectrums. Lastly, we suggest further efforts to understand whether and how to counter the role of system justification within environmental challenges.

## Additional File

The additional file for this article can be found as follows:

10.5334/irsp.871.s1 Supplementary Materials. Additional methods details and supplementary results.

## Footnotes

Specifically, these data report descriptive statistics on Kay and Jost’s (2003) system justification measure, which ranges from 1 to 9. For the 2010 fall semester: *M* = 4.53, *SD* = 1.28; 2011 spring semester: *M* = 4.41, *SD* = 1.17; 2011 fall semester: *M* = 4.49, *SD* = 1.57; 2012 spring semester: *M* = 4.48, *SD* = 1.18. See also data collected in 2007 and published in van der Toorn et al. (2010; *M* = 4.45, *SD* = 1.22).

The survey was initially posted as a four-minute survey, and payment increased accordingly after responses from the first 50 participants demonstrated we underestimated the survey length. This remuneration is consistent with studies administered through Prolific, though differs from the original study, which awarded course credit in exchange for participation.

See Supplementary Materials for analyses with participants who were flagged as inattentive *(n* = 59) and non-naïve (*n* = 67) removed, which show the same findings.

We deviated from our pre-registration when analysing the total petition scores because Poisson and negative binomial regressions are better methods for analysing count data than ordinary regression analysis (see Coxe et al. 2009). Of the two, analysis of residuals indicated better fit to the data for the negative binomial model, hence we present results from the negative binomial analyses here.

## References

[1] Adler, N. E., Epel, E. S., Castellazzo, G., & Ickovics, J. R. (2000). Relationship of subjective and objective social status with psychological and physiological functioning: Preliminary data in healthy, White women. Health Psychology, 19(6), 586–592. DOI: 10.1037/0278-6133.19.6.586 11129362

[2] Baldwin, M., & Lammers, J. (2016). Past-focused environmental comparisons promote proenvironmental outcomes for conservatives. Proceedings of the National Academy of Sciences, 113(52), 14953–14957. DOI: 10.1073/pnas.1610834113 PMC5206530 27956619

[3] Berkebile-Weinberg, M., Goldwert, D., Doell, K. C., Van Bavel, J. J., & Vlasceanu, M. (2024). The differential impact of climate interventions along the political divide in 60 countries. Nature Communications, 15(1), 3885. DOI: 10.1038/s41467-024-48112-8 PMC11078920 38719845

[4] Bernauer, T., & McGrath, L. F. (2016). Simple reframing unlikely to boost public support for climate policy. Nature Climate Change, 6, 680–683. DOI: 10.1038/nclimate2948

[5] Campbell, T. H., & Kay, A. C. (2014). Solution aversion: On the relation between ideology and motivated disbelief. Journal of Personality and Social Psychology, 107(5), 809–824. DOI: 10.1037/a0037963 25347128

[6] Cichocka, A., & Jost, J. T. (2014). Stripped of illusions? Exploring system justification processes in capitalist and post-communist societies. International Journal of Psychology, 49(1), 6–29. DOI: 10.1002/ijop.12011 24811719

[7] Clarke, E. J., Ling, M., Kothe, E. J., Klas, A., & Richardson, B. (2019). Mitigation system threat partially mediates the effects of right-wing ideologies on climate change beliefs. Journal of Applied Social Psychology, 49(6), 349–360. DOI: 10.1111/jasp.12585

[8] Clayton, S. (2020). Climate anxiety: Psychological responses to climate change. Journal of Anxiety Disorders, 74, Article 102263. DOI: 10.1016/j.janxdis.2020.102263 32623280

[9] Clifford, S., & Jerit, J. (2014). Is there a cost to convenience? An experimental comparison of data quality in laboratory and online studies. Journal of Experimental Political Science, 1(2), 120–131. DOI: 10.1017/xps.2014.5

[10] Coxe, S., West, S. G., & Aiken, L. S. (2009). The analysis of count data: A gentle introduction to Poisson regression and its alternatives. Journal of Personality Assessment, 91(2), 121–136. DOI: 10.1080/00223890802634175 19205933

[11] Diamond, E. P. (2020). The influence of identity salience on framing effectiveness: An experiment. Political Psychology, 41(6), 1133–1150. DOI: 10.1111/pops.12669

[12] Feygina, I., Jost, J. T., & Goldsmith, R. E. (2010). System justification, the denial of global warming, and the possibility of ‘system-sanctioned change’. Personality and Social Psychology Bulletin, 36(3), 326–338. DOI: 10.1177/0146167209351435 20008965

[13] Harring, M., & Sohlberg, J. (2016). The varying effects of left–right ideology on support for the environment: Evidence from a Swedish survey experiment. Environmental Politics, 26(2), 278–300. DOI: 10.1080/09644016.2016.1244965

[14] Hart, P. S., & Nisbet, E. C. (2011). Boomerang effects in science communication: How motivated reasoning and identity cues amplify opinion polarization about climate mitigation policies. Communication Research, 39(6), 701–723. DOI: 10.1177/0093650211416646

[15] Hennes, E. P., Ruisch, B. C., Feygina, I., Monteiro, C. A., & Jost, J. T. (2016). Motivated recall in the service of the economic system: The case of anthropogenic climate change. Journal of Experimental Psychology: General, 145(6), 755–771. https://psycnet.apa.org/doi/10.1037/xge0000148 27123575 10.1037/xge0000148

[16] Hughes, J. (2017). Paramtest: Run a function iteratively while varying parameters (0.1.0) [Computer software]. https://CRAN.R-project.org/package=paramtest

[17] Jost, J. T., & Banaji, M. R. (1994). The role of stereotyping in system-justification and the production of false consciousness. British Journal of Social Psychology, 33(1), 1–27. DOI: 10.1111/j.2044-8309.1994.tb01008.x

[18] Jost, J. T., Banaji, M. R., & Nosek, B. A. (2004). A decade of system justification theory: Accumulated evidence of conscious and unconscious bolstering of the status quo. Political Psychology, 25(6), 881–919. DOI: 10.1111/j.1467-9221.2004.00402.x

[19] Jost, J. T., Becker, J., Osborne, D., & Badaan, V. (2017). Missing in (collective) action: Ideology, system justification, and the motivational antecedents of two types of protest behavior. Current Directions in Psychological Science, 26(2), 99–108. DOI: 10.1177/0963721417690633

[20] Jost, J. T., Chaikalis-Petritsis, V., Abrams, D., Sidanius, J., Van Der Toorn, J., & Bratt, C. (2012). Why men (and women) do and don’t rebel: Effects of system justification on willingness to protest. Personality and Social Psychology Bulletin, 38(2), 197–208. DOI: 10.1177/0146167211422544 21911420

[21] Jost, J., & Hunyady, O. (2002). The psychology of system justification and the palliative function of ideology. European Review of Social Psychology, 13(1), 111–153. DOI: 10.1080/10463280240000046

[22] Jost, J. T., Liviatan, I., Van Der Toorn, J., Ledgerwood, A., Mandisodza, A., & Nosek, B. A. (2011). System justification: How do we know it’s motivated? In *The psychology of justice and legitimacy* (pp. 173–203). Psychology Press.

[23] Jylhä, K. M. (2016). Ideological roots of climate change denial: Resistance to change, acceptance of inequality, or both? Doctoral thesis, Uppsala University, Uppsala, Sweden.

[24] Jylhä, K. M., & Akrami, N. (2015). Social dominance orientation and climate change denial: The role of dominance and system justification. Personality and Individual Differences, 86, 108–111. DOI: 10.1016/j.paid.2015.05.041

[25] Kay, A. C., & Friesen, J. (2011). On social stability and social change: Understanding when system justification does and does not occur. Current Directions in Psychological Science, 20(6), 360–364. DOI: 10.1177/0963721411422059

[26] Kay, A. C., Gaucher, D., Peach, J. M., Laurin, K., Friesen, J., Zanna, M. P., & Spencer, S. J. (2009). Inequality, discrimination, and the power of the status quo: Direct evidence for a motivation to see the way things are as the way they should be. Journal of Personality and Social Psychology, 97(3), 421–434. https://psycnet.apa.org/doi/10.1037/a0015997 19685999 10.1037/a0015997

[27] Kay, A. C., & Jost, J. T. (2003). Complementary justice: Effects of ‘poor but happy’ and ‘poor but honest’ stereotype exemplars on system justification and implicit activation of the justice motive. Journal of Personality and Social Psychology, 85(5), 823–837. https://psycnet.apa.org/doi/10.1037/0022-3514.85.5.823 14599247 10.1037/0022-3514.85.5.823

[28] Kim, I., Hammond, M. D., & Milfont, T. L. (2021). Do past-focused environmental messages promote pro-environmentalism to conservatives? A pre-registered replication. Journal of Environmental Psychology, 73, Article 101547. DOI: 10.1016/j.jenvp.2020.101547

[29] Kim, I., Hammond, M. D., & Milfont, T. L. (2023). Do environmental messages emphasising binding morals promote conservatives’ pro-environmentalism? A pre-registered replication. Social Psychological Bulletin, 18, 1–24. DOI: 10.32872/spb.8557

[30] Lange, F., & Dewitte, S. (2019). Measuring pro-environmental behavior: Review and recommendations. Journal of Environmental Psychology, 63, 92–100. DOI: 10.1016/j.jenvp.2019.04.009

[31] Lange, F., & Dewitte, S. (2022). The work for environmental protection task: A consequential web-based procedure for studying pro-environmental behavior. Behavior Research Methods, 54(1), 133–145. DOI: 10.3758/s13428-021-01617-2 34109560

[32] Lenth, R. V. (2023). Emmeans: Estimated marginal means, aka least-squares means (1.8.5) [Computer software]. https://CRAN.Rproject.org/package=emmeans

[33] Long, J. A. (2021). Interactions: Comprehensive, user-friendly toolkit for probing interactions (1.1.5) [Computer software]. https://cran.r-project.org/package=interactions

[34] Marquardt, J., & Nasiritousi, N. (2022). Imaginary lock-ins in climate change politics: The challenge to envision a fossil-free future. Environmental Politics, 31(4), 621–642. DOI: 10.1080/09644016.2021.1951479

[35] Ojala, M., Cunsolo, A., Ogunbode, C. A., & Middleton, J. (2021). Anxiety, worry, and grief in a time of environmental and climate crisis: A narrative review. Annual Review of Environment and Resources, 46, 35–58. https://www.annualreviews.org/doi/abs/10.1146/annurev-environ-012220-022716

[36] Osborne, D., Sengupta, N. K., & Sibley, C. G. (2018). System justification theory at 25: Evaluating a paradigm shift in psychology and looking towards the future. British Journal of Social Psychology, 58(2), 340–361. DOI: 10.1111/bjso.12302 30525206

[37] Peer, E., Rothschild, D., Gordon, A., Evernden, Z., & Damer, E. (2021). Data quality of platforms and panels for online behavioral research. Behavior Research Methods, 54, 1643–1662. DOI: 10.3758/s13428-021-01694-3 34590289 PMC8480459

[38] Peters, G.-J. Y., & Gruijters, S. (2021). ufs: A collection of utilities (0.4.5) [Computer software]. https://r-packages.gitlab.io/ufs

[39] Pihkala, P. (2020). Anxiety and the ecological crisis: An analysis of eco-anxiety and climate anxiety. Sustainability, 12(19), 7836. DOI: 10.3390/su12197836

[40] R Core Team. (2022). R: A language and environment for statistical computing. R for statistical computing (4.2.1) [Computer software]. https://www.R-project.org

[41] R Core Team. (2023). R: A language and environment for statistical computing. R for statistical computing (4.3.0) [Computer software]. https://www.R-project.org

[42] Ritchie, H. (2019). Who has contributed most to global CO_2_ emissions? Our World in Data. Retrieved from: https://ourworldindata.org/contributed-most-global-co2

[43] Sassenberg, K., & Ditrich, L. (2019). Research in social psychology changed between 2011 and 2016: Larger sample sizes, more self-report measures, and more online studies. Advances in Methods and Practices in Psychological Science, 2(2), 107–114. DOI: 10.1177/2515245919838781

[44] Sotola, L. K., & Credé, M. (2022). On the predicted replicability of two decades of experimental research on system justification: AZ-curve analysis. European Journal of Social Psychology, 52(5–6), 895–909. DOI: 10.1002/ejsp.2858

[45] Stanley, S. K., Hogg, T. L., Leviston, Z., & Walker, I. (2021b). From anger to action: Differential impacts of eco-anxiety, eco-depression, and eco-anger on climate action and wellbeing. The Journal of Climate Change and Health, 1, Article 100003. DOI: 10.1016/j.joclim.2021.100003

[46] Stanley, S. K., Klas, A., Clarke, E. J., & Walker, I. (2021a). The effects of a temporal framing manipulation on environmentalism: A replication and extension. PLoS ONE, 16(2), Article e0246058. DOI: 10.1371/journal.pone.0246058 PMC7877654 33571222

[47] Stroebe, W., & Strack, F. (2014). The alleged crisis and the illusion of exact replication. Perspectives on Psychological Science, 9(1), 59–71. DOI: 10.1177/1745691613514450 26173241

[48] van der Toorn, J., Berkics, M., & Jost, J. T. (2010). System justification, satisfaction, and perceptions of fairness and typicality at work: A cross-system comparison involving the US and Hungary. Social Justice Research, 23, 189–210. DOI: 10.1007/s11211-010-0116-1

[49] Van Zomeren, M., Postmes, T., & Spears, R. (2008). Toward an integrative social identity model of collective action: a quantitative research synthesis of three socio-psychological perspectives. Psychological Bulletin, 134(4), 504–535. DOI: 10.1037/0033-2909.134.4.504 18605818

[50] Vargas-Salfate, S., Paez, D., Liu, J. H., Pratto, F., & Gil de Zúñiga, H. (2018). A comparison of social dominance theory and system justification: The role of social status in 19 nations. Personality and Social Psychology Bulletin, 44(7), 1060–1076. DOI: 10.1177/0146167218757455 29544389

[51] Venables, W. N., & Ripley, B. D. (2002). Modern applied statistics with S (4th ed.). Springer. https://www.stats.ox.ac.uk/pub/MASS4/

[52] Vlasceanu, M., Doell, K. C., Bak-Coleman, J. B., Todorova, B., Berkebile-Weinberg, M. M., Grayson, S. J., … & Lutz, A. E. (2024). Addressing climate change with behavioral science: A global intervention tournament in 63 countries. Science Advances, 10(6), Article eadj5778. DOI: 10.1126/sciadv.adj5778 PMC10849597 38324680

[53] Wickham, H. (2016). Ggplot2: Elegant graphics for data analysis (3.5.0) [Computer software]. https://ggplot2.tidyverse.org

[54] Wullenkord, M. C. (2022). From denial of facts to rationalization and avoidance: Ideology, needs, and gender predict the spectrum of climate denial. Personality and Individual Differences, 193, Article 111616. DOI: 10.1016/j.paid.2022.111616

[55] Wullenkord, M. C., Tröger, J., Hamann, K. R., Loy, L. S., & Reese, G. (2021). Anxiety and climate change: A validation of the Climate Anxiety Scale in a German-speaking quota sample and an investigation of psychological correlates. Climatic Change, 168(3–4), Article 20. DOI: 10.1007/s10584-021-03234-6

