# Supplementary material for: Limited and Mixed Evidence for System-Sanctioned Change to Protect the Environment: A Replication Study
Source: Int Rev Soc Psychol. 2024 Aug 30;37:16. doi: 10.5334/irsp.871 (PMC12372673; doi:10.5334/irsp.871)
Supplement: Supplementary Materials. — Additional methods details and supplementary results. [file irsp-37-871-s1.pdf]

Supplementary Materials

Additional methods details

Comprehension questions

The instructions for the comprehension questions were those used in the original study: “Here we are interested in your thoughts about the passages that you read — people are considering using them on a website, so we would like your opinion about their ease of readability. Please rate your agreement or disagreement with each of the following statements using the scale below.” Participants were asked “to what extent were these passages...” clear, easy to understand, interesting, and persuasive, from 1 (not at all) to 7 (extremely).

Attention checks

The in-person nature of the experiment gave the researchers more experimental control and oversight of participants. To ensure a similar level of data quality in the online, anonymous setting, we included several additional checks at the end of the experiment to identify less engaged participants. The first was time spent on the survey (automatically captured in Qualtrics), and the second was a multiple-choice question: “The color test you are about to take part in is very simple, when asked for your favorite color you must select ‘Orange’. This is an attention check. Based on the text you read above, what color have you been asked to enter?”.

Naivety checks

We included two yes/no naivety checks: “Do you remember taking part in a similar study before?”, and “Have you ever heard about research indicating people who prefer the status quo find messages describing environmental protection as preserving the American way of life more persuasive?”.

Supplementary results

To further investigate petition signing, we compared the percentage of participants interested in signing most petitions across conditions. For those higher in system justification, 49.33% wished to sign most petitions in the system-preservation condition, which is an increase from 34.40% in the original study control condition. However, for those lower (*M* − 1*SD*) in system justification, more participants wanted to sign most petitions in the original study control condition (60.59%) than in the system-preservation condition (47.48%). Full results are shown in Table S1, and suggest this pattern continues at more extreme levels of system justification (above and below 1.5SD from the mean). This analytical method, which was modelled on the original study, has two limitations: it only focused on percentages signing ‘most’ petitions, and it does not indicate whether the discrepancies of those percentages are statistically significant or not. Follow up analyses of those with higher tendencies to justify the system (*M* + 1*SD*) did not find a significant difference between the number of petitions signed in the original study control (*M* = 2.41, *SE* = 0.31) or system-preservation condition (*M* = 3.15, *SE* = 0.36), or a difference in the number of petitions signed by those with low levels of system justification (*M* − 1*SD*) assigned to the original study condition (*M* = 4.06, *SE* = 0.47) versus the system-preservation condition (*M* = 3.19, *SE* = 0.39).

Table S1.

Percentages of those low and high in system justification signing none, a few, and most environmental petitions in the original study conditions.

	**Lower system justification**		**Higher system justification**	
	M – 1.5*SD*	*M* – 1*SD*	*M* + 1*SD*	*M* + 1.5*SD*
**Original study control**				
None	18.56	22.97	46.66	53.37
A few	14.64	16.43	18.95	18.02
Most	66.80	60.59	34.40	28.61
**System-preservation condition**				
None	34.07	33.66	32.02	31.62
A few	18.91	18.86	18.65	18.59
Most	47.02	47.48	49.33	49.79

Note. Percentages in the original study conditions differ from Table 5 because Table 5 includes the full sample of data, and thus *M* and *SD* differ slightly.

Effects with conservative political orientation

Here, we elaborate on findings mentioned in section "Effects with conservative political orientation" of the main text.

We found no significant interaction between political orientation and the original study conditions (i.e., the original control condition and the system-preservation condition) on PEB intentions ($b = -0.01$, $SE = .01$, $p = .363$) in an analysis only with the two conditions. Nor did we find any evidence that the effect of political orientation on PEB intentions differed when comparing the true control group participants with those in the system-preservation condition ($b = 0.0008$, $p = .901$) or the original study control condition ($b = 0.01$, $p = .276$). Moreover, when considering petition-signing intentions, an ordinal logistic regression analysis with the two conditions showed that the interaction effect between political orientation and the system-preservation framing condition (versus the original condition) was not significant, $b = 0.01$, SE = .01, odds ratio (OR) = 1.01, $p = .250$. Also, in another ordinal logistic regression analysis with all the conditions, there were no significant interaction effects between political orientation when comparing the original and true control conditions ($b = -0.01$, OR = 0.99, $p = .450$) or system-preservation and true control conditions ($b < 0.01$, OR = 1.00, $p = .652$).

Results of analyses with exclusion criteria applied ($N = 447$)

In our preregistration, we specified that we would run analyses on the full sample and then again to report any differences in results when we remove those who fail the attention check, complete the study in less than half the median completion time, or indicate that they are familiar with the study (i.e., respond 'yes' to either of our two naivety checks). Interestingly, no participants failed the attention check, though 59 were identified as inattentive based on their survey completion time. Of our naivety checks, 36 recalled taking part in a similar study and 38 had heard about research indicating people who prefer the status quo find messages describing environmental protection as preserving the American way of life more persuasive. As these groups overlapped, we identified 67 non-naïve participants in total. We conducted the same analyses on a sample in which inattentive and non-naive people were excluded ($N = 447$). The overall patterns of results do not differ from the results based on our full sample reported in the main text.

Demographics

Participants had a more restricted age range compared to the full sample (18 to 79 years), though very similar mean and standard deviation ($M = 37.80$, $SD = 13.72$). Similar to the full sample, 45.64% identified as men and 51.01% as women, and the majority reported their race as White (77.40%). The remainders are Black or African American (7.61%). Asian (6.94%), Some other race (4.47%), Prefer not to say (1.57%), American Indian or Alaska Native (1.34%), and

Native Hawaiian or Other Pacific Islander (0.67%). The sample leaned liberal (*M* = 34.13, *SD* = 27.92).

Effects on pro-environmental intentions

Two conditions. Regressing pro-environmental behavioural intentions on system justification, experimental condition (including only the original study conditions: system-preservation framing condition versus control condition), and their interaction, identified no significant effects of system justification ($b = 0.03$, $p = .760$), condition ($b = -0.30$, $p = .588$), or their interaction ($b = 0.02$, $p = .863$) on PEB intentions.

Three conditions. We found a significant interaction between system justification and condition, whereby the effect of system justification on PEB intentions differs between those in the original and true control condition ($b = 0.30$, $p = .028$), and between those in the system-preservation experimental condition and true control condition ($b = 0.33$, $p = .015$). Following up the significant interactions, simple slope analysis showed a significant negative relationship between system justification and PEB intentions only in the true control condition ($b = -0.27$, $p = .004$), while the association was non-significant in both the original study control condition ($b = 0.03$, $p = .759$) and system-preservation condition ($b = 0.05$, $p = .568$). When we compared PEB intentions at low ($M - 1SD$) and high ($M + 1SD$) levels of system justification in each condition, we found significantly *lower* PEB intentions for low system justifiers in the system preservation condition ($M = 4.27$, $SE = 0.22$) than the true control condition ($M = 4.95$, $SE = 0.21$), $t(441) = -2.23$, $p = .0495$. There were no other significant differences (remaining *p's* > .166).

Effects on signing petitions

Two conditions. The analysis using the ordinal petition variable revealed a significant interaction between system justification and condition ($b = 0.44$, $SE = 0.15$, 95% CI [0.15, 0.73], OR = 1.55, $p = .003$). Simple slope analyses found a statistically significant negative effect of system justification on the likelihood of signing petitions in the original study control condition ($b = -0.38$, $SE = 0.11$, 95% Asymptotic CI [−0.59, −0.16], $p < .001$), and no effect of system justification on petition signing within the system-preservation condition *(*$b = 0.07$, SE = 0.10, 95% Asymptotic CI [−0.13, 0.26], $p = .507$). For those low in system justification, 63.11% wished to sign most petitions in the original study control condition, and 47.08% of those in the system-preservation condition. For those high in system justification, 35.52% wished to sign most petitions in the original study control condition, compared to 52.07% in the system-preservation condition.

Three conditions. Again consistent with the analyses reported in the main text, the extent to which system justification predicted signing none, a few, or most petitions did not differ between original and true control conditions ($b = -0.09$, $SE = 0.15$, 95% CI [−0.38, 0.20],

OR = 0.91, *p* = .547), however it did differ between true control and system-preservation conditions (*b* = 0.35, *SE* = 0.14, 95% CI [0.07, 0.63], OR = 1.41, *p* = .014). The simple slope analysis showed significant and negative relationships between system justification and petition signing in both the original (*b* = −0.37, $p < .001$) and true control conditions (*b* = −0.28, *p* = .005), and no significant association in the system-preservation condition (*b* = 0.07, *p* = .511). In the true control condition, most participants low in system justification were willing to sign most petitions (63.67% of the sample, versus 41.96% of those high in system justification), similar to the original control condition (64.08% of low system justifiers, versus 35.75% of those high in system justification). By contrast, in the preservation framing condition, there was little difference in the percentages of signing most petitions between participants low and high in system justification (47.35% versus 52.47%, respectively).

Effects with conservative political orientation

Political orientation did not significantly interact with original study conditions (i.e., the original control condition and the preservation framing condition) to affect PEB intentions (*b* = −0.004, *p* = .618). There was also no significant interaction effect on PEB intentions as predicted by political orientation and true control versus system-preservation condition interaction (*b* = 0.001, *p* = .868) or the original study control condition (*b* = 0.005, *p* = .495). Regarding petition-signing intentions, an ordinal logistic regression analysis found that the interaction effect between political orientation and the system-preservation framing condition (versus the original condition) was not statistically significant, *b* = 0.01, *p* = .094. Repeating this analysis with all conditions also returned no statistically significant interaction effects between political orientation and both conditions (versus the true control condition), *bs* = −0.01 to 0.01, $ps \geq .330$.

Conclusions

These additional findings show that the same results reported in text hold when participants who completed the study with a very fast completion time or were not naïve to the experiment were retained.
